# The safety of live-attenuated vaccines in patients using IL-1 or IL-6 blockade: an international survey

**DOI:** 10.1186/s12969-018-0235-z

**Published:** 2018-03-21

**Authors:** Jerold Jeyaratnam, Nienke M. ter Haar, Helen J. Lachmann, Ozgur Kasapcopur, Amanda K. Ombrello, Donato Rigante, Fatma Dedeoglu, Ezgi H. Baris, Sebastiaan J. Vastert, Nico M. Wulffraat, Joost Frenkel

**Affiliations:** 1 Department of General Pediatrics, University Medical Center Utrecht, Room KE 04 133 1, PO-Box 85090, 3508, AB Utrecht, The Netherlands; 2 Laboratory for Translational Immunology, University Medical Center Utrecht, Utrecht, the Netherlands; 3 University College Medical School, National Amyloidosis Center, Royal Free Campus, London, UK; 4 Department of Pediatric Rheumatology, Cerrahpasa Medical School-Istanbul University, Istanbul, Turkey; 5 Inflammatory Disease section, National Human Genome Research Institute, Bethesda, MA USA; 6 Institute of Pediatrics, Università Cattolica Sacro Cuore, Rome, Italy; 7 Division of Immunology, Boston Children’s Hospital, Boston, MA USA; 8 Department of Pediatric Rheumatology, University Medical Center Utrecht, Utrecht, the Netherlands

**Keywords:** Autoinflammatory diseases, Live-attenuated vaccines, Biologicals, IL-1 blockade, IL-6 blockade

## Abstract

**Background:**

Withholding live-attenuated vaccines in patients using interleukin (IL)-1 or IL-6 blocking agents is recommended by guidelines for both pediatric and adult rheumatic diseases, since there is a risk of infection in an immune suppressed host. However, this has never been studied. This retrospective, multicenter survey aimed to evaluate the safety of live-attenuated vaccines in patients using IL-1 or IL-6 blockade.

**Methods:**

We contacted physicians involved in the treatment of autoinflammatory diseases to investigate potential cases. Patients were included if a live-attenuated vaccine had been administered while they were on IL-1 or IL-6 blockade.

**Results:**

Seventeen patients were included in this survey (7 systemic juvenile idiopathic arthritis (sJIA), 5 cryopyrin associated periodic syndrome (CAPS), 4 mevalonate kinase deficiency (MKD) and 1 familial Mediterranean fever (FMF). Three patients experienced an adverse event, of which two were serious adverse events (a varicella zoster infection after varicella zoster booster vaccination, and a pneumonia after MMR booster). One additional patient had diarrhea after oral polio vaccine. Further, seven patients experienced a flare of their disease, which were generally mild. Eight patients did not experience an adverse event or a flare.

**Conclusion:**

We have described a case series of seventeen patients who received a live-attenuated vaccine while using IL-1 or IL-6 blocking medication. The findings of this survey are not a reason to adapt the existing guidelines. Prospective trials are needed in order to acquire more evidence about the safety and efficacy before considering adaptation of guidelines.

## Background

Vaccines have contributed greatly to public health, protecting children and adults from serious infectious diseases [1]. Besides inactivated vaccines there are several live-attenuated vaccines.

Autoinflammatory diseases, such as systemic juvenile idiopathic arthritis (sJIA), familial Mediterranean fever (FMF), mevalonate kinase deficiency (MKD), cryopyrin associated periodic syndrome (CAPS) and tumor necrosis factor (TNF)-receptor associated periodic syndrome (TRAPS) exhibit systemic inflammation or organ specific inflammation caused by dysregulation of the innate immune system. Interleukin (IL)-1 blockade (anakinra, canakinumab and rilonacept) and IL-6 blockade (tocilizumab) have greatly improved the outcome of these patients [2]. However, patients with autoinflammatory diseases potentially face long-term or lifelong immunosuppression thus raising the dilemma if and when vaccinations could be given.

Although vaccination is generally considered safe in the healthy population, existing guidelines for both pediatric and adult rheumatic diseases recommend to withhold live-attenuated vaccines in patients using IL-1 or IL-6 blocking agents, because of lack of safety data and the (theoretical) risk of introducing infection in an immune suppressed host [3–5]. On the other hand, patients with autoinflammatory diseases might especially benefit from protection against infectious diseases, as the immunosuppressive therapy renders them more susceptible to infections [6, 7]. In clinical practice, vaccination can be considered on a case-to-case basis weighing the risk of natural infection versus the risk of vaccination, e.g. measles mumps and rubella (MMR) vaccination during measles outbreaks or yellow fever vaccine before travelling to endemic regions.

In this retrospective survey, we aimed to evaluate the safety and efficacy of live-attenuated vaccines in patients using IL-1 or IL-6 blockade.

## Methods

Via the Paediatric Rheumatology European Society and the International Society for Systemic Auto Inflammatory Diseases pediatric and adult rheumatologists and immunologists were contacted by e-mail in January 2016 in order to recruit potential cases. A reminder was sent up to three times, if no response was obtained. Patients were included if they had received a live-attenuated vaccine while using IL-1 blockade or IL-6 blockade.

Ethical approval was obtained by the institutional ethical committee in accordance with local ethical regulations; the survey was conducted in accordance with the ethical principles of the declaration of Helsinki. Written informed consent was obtained from the patient or the legal guardians in case of minors according to local requirements. Demographic and clinical data were collected by local physicians and were anonymized. Adverse events were categorized as adverse events and serious adverse events. Serious adverse events were defined as events leading to death, life-threatening events, events leading to hospitalization, prolonged hospitalization and events leading to severe and/or permanent disability [8]. A flare was defined as the presence of any symptoms of the disease, e.g. fever, rash, abdominal pain and diarrhea.

## Results

In total 85 physicians from 23 countries were contacted. In total, seventeen patients were included: 7 sJIA, 5 CAPS, 4 MKD and 1 FMF. The median age at vaccination was 9 years (1–58 years), 11 patients were female. Most patients received a booster vaccine for MMR, but patients also received varicella zoster, yellow fever and oral polio vaccines. Patient characteristics are listed in Table 1. The patients are discussed in more detail below.

**Table 1:** Characteristics of all vaccinated patients

Pt	Gender	Age (y)	Country	Disease	Disease activity	Biological	Other DMARDs	Vaccination	Adverse events	Flare
1	Female	7	USA	sJIA	Inactive	Anakinra 4 mg/kg/day	Prednisone (0.12 mg/kg/day) Methotrexate Leflunomide Thalidomide	Varicella zoster (booster)	Varicella zoster infection	–
2	Female	1	Turkey	sJIA	Inactive	Canakinumab 4 mg/kg/2 months	Prednisone (5 mg/day) Methotrexate	MMR booster	Pneumonia	Yes
3	Male	2	Turkey	sJIA	Inactive	Tocilizumab 12 mg/kg/monthly	Prednisone (2.5 mg/day)	Oral polio vaccine	Diarrhea	–
4	Male	12	USA	MKD	Inactive	Anakinra^a^ 100 mg/day (STOP 3-4D-1D)	–	Varicella zoster (booster)	–	Yes
5	Male	9	USA	MKD	Inactive	Anakinra^a^ 150 mg during flare (STOP 2D-1D)	–	Varicella zoster (booster)	–	Yes
6	Female	4	USA	MKD	Partially active	Canakinumab^a^ 4 mg/kg/6 weeks (STOP 3 M–3 M)		Varicella zoster (first vaccine) and MMR first vaccine	–	Yes
7	Female	2	Turkey	FMF	Inactive	Canakinumab 4 mg/kg/monthly	Prednisone (2.5 mg/day) Colchicine	MMR booster	–	Yes
8	Female	9	Netherlands	sJIA	Inactive	Anakinra^a^ 1.7 mg/kg/2 days (STOP 2D-3D)	Methotrexate	MMR booster	–	Yes
9	Male	3	Netherlands	sJIA	Inactive	Tocilizumab 132 mg/14 days)	Prednisone (2.5 mg/day)	Varicella zoster (first vaccine)	–	Yes
10	Male	12	Italy	CAPS	Inactive	Anakinra^a^ 1.5 mg/kg/day (STOP 3D-14D)	Methylprednisolone (0.06 mg/kg/day)	MMR booster	–	–
11	Female	12	Italy	MKD	Partially active	Anakinra^a^ 1.4 mg/kg/day (STOP 3D-28D)	–	MMR booster	–	–
12	Female	9	Netherlands	sJIA	Inactive	Anakinra 40 mg/3 days	–	MMR booster	–	–
13	Male	9	Netherlands	sJIA	Inactive	Tocilizumab 8 mg/kg/2 weeks	Methotrexate	MMR booster	–	–
14	Female	58	United Kingdom	CAPS	Inactive	Anakinra^a^ 100 mg/day (STOP 3D-3D)	–	Yellow fever (first vaccine)	–	–
15	Female	28	United Kingdom	CAPS	Inactive	Anakinra^a^ 100 mg/day (STOP 3D-3D)	–	Yellow fever (first vaccine)	–	–
16	Female	26	United Kingdom	CAPS	Inactive	Anakinra^a^ 100 mg/day (STOP 3D-3D)	–	Yellow fever (first vaccine)	–	–
17	Female	44	United Kingdom	CAPS	Inactive	Canakinumab (150 mg/2 months)	–	Yellow fever (first vaccine)	–	–

^a^Biological stopped prior to vaccination and restarted after vaccination. The period of discontinuation before and after vaccination is indicated in brackets.

*D* days before or after vaccination, *M* months before or after vaccination

### Adverse events

Three patients reported an adverse event after vaccination, of whom two were categorized as severe due to the need for hospitalization.

Patient 1 was a seven-year-old girl with sJIA, who was treated with multiple immunosuppressive agents at the same time, including prednisone, methotrexate (MTX), thalidomide, leflunomide and anakinra. Sixteen days after she received a varicella zoster booster vaccination, she developed vesicles on her trunk and a few on her scalp and extremities. The diagnosis of varicella zoster infection was made on the clinical phenotype; the virus could not be isolated. Because of suspected varicella in an immunocompromised host, she was admitted to hospital for 4 days and was discharged with a 10-day course of intravenous acyclovir. As there was no known exposure to other varicella cases, the infection in this child was considered to be caused by the vaccination itself.

Patient 2 suffered from sJIA and was treated with canakinumab. She was accidentally vaccinated with MMR booster by the vaccination campaign in Turkey. One week after vaccination she was diagnosed with pneumonia, which was confirmed by X-ray. As it was considered to be a bacterial pneumonia, treatment with cefuroxime axetil was started. The patient was hospitalized for 5 days. Besides the pneumonia, she had a sJIA flare with fever and rash during the same period. This flare was treated with low-dose prednisone with good response. The impression of the physicians in charge was that this was a flare of her sJIA. Although extremely unlikely, the coincidence of fever, rash and pneumonia could also be ascribed to measles, introduced by vaccination.

The last patient (patient 3) with an adverse event was a sJIA patient treated with tocilizumab. One week after vaccination with oral polio vaccine, the patient experienced diarrhea, which was treated with oral fluid replacements, co-trimoxazol and probiotics. This adverse event was thought to be caused by the vaccine, especially since other family members did not suffer from diarrhea.

### Flares

In total seven patients reported a flare of their disease after vaccination. A MKD patient (patient 4) received 100 mg anakinra daily, which was stopped 3–4 days before booster vaccination with varicella zoster and restarted 1 day after vaccination. He experienced fever after vaccination. Since the fever was thought to be caused by a flare, the patient was treated with the normal dose of anakinra. Patient 5 used anakinra (150 mg) during MKD flares only, since the disease was relatively quiescent. The days before and after vaccination with varicella zoster, anakinra was not administered. After vaccination he experienced fever, which was treated with anakinra. Patient 6 was 4 years old when she received her first MMR and varicella zoster vaccines. Her disease was incompletely controlled under treatment with canakinumab 4 mg/kg every 6 weeks. Canakinumab was stopped 3 months before and restarted 3 months after vaccination. Before vaccination she experienced low grade fevers every week. After vaccination she had a mild flare with fever, vomiting, diarrhea and headache. This flare was treated with acetaminophen and ibuprofen. Patient 7 was treated with canakinumab due to colchicine resistant FMF. Besides FMF, she also suffered from inflammatory bowel disease. The vaccination with MMR booster was administered accidentally by the primary health service in Turkey. Due to this vaccination the next dose of canakinumab was withheld; the following dose was given 2 months after vaccination. One week after vaccination the patient had an FMF attack with fever and abdominal pain, which led to hospitalization. During hospitalization she was treated with low-dose prednisone (2.5 mg/day) and colchicine (0.5 mg/day) with good response. After 8 days she was discharged in good condition.

In another patient (patient 8) who suffered from sJIA, anakinra was stopped 2 days before and restarted 3 days after vaccination. After MMR booster vaccination she experienced a mild flare with some exanthema, but without fever, which was probably due to the stop of anakinra. This flare did not require any additional treatment.

Patient 9 was a young boy with Down syndrome and sJIA complicated by macrophage activation syndrome. He was initiated on tocilizumab treatment at a very young age, before experiencing varicella zoster infection; therefore, he was vaccinated with a first varicella zoster vaccine, in order to acquire immunity in a controlled setting. After vaccination he suffered from a mild flare with subfebrile temperature, exanthema and malaise. After this flare, the physician and parents decided not to repeat varicella vaccination.

### Patients without flares or adverse events

Eight patients did not experience a flare or an adverse event. Patient 10 suffered from Chronic Infantile Neurological Cutaneous Articular (CINCA) syndrome, the most severe form of CAPS, which was treated with anakinra. At the age of 12 years he received MMR vaccination as a routine administration of the booster vaccine. Treatment with anakinra was stopped 3 days before vaccination and restarted 2 weeks afterwards. He did not have any adverse events. Patient 11 who had MKD was in partial remission at the time of vaccination. The treatment of anakinra was stopped 3 days before vaccination and restarted 4 weeks afterwards. The last two patients were Dutch sJIA patients with inactive disease on anakinra (patient 12) or tocilizumab and MTX (patient 13). They received the MMR booster vaccine through the national vaccination program, and reported no symptoms afterwards. Patients 14–16 concerned a mother and two daughters all suffering from CAPS, which was treated with anakinra. In order to be protected against yellow fever during travel, they were all vaccinated without any problems. The three of them suspended anakinra for 3 days prior and 3 days after vaccination. The last patient (patient 17) was treated with canakinumab and received a yellow fever vaccination. The vaccine was given 8 weeks after the last dose and the next dose was given 3 weeks after vaccination.

## Discussion

This survey describes the safety of live-attenuated vaccine in a case series of patients using IL-1 or IL-6 blocking medication. The seventeen patients in our series reported three adverse events, of which two were categorized as severe, and seven flares, while eight patients did not report any complaints after vaccination. Although adverse events occurred soon after vaccination, coincidence cannot be ruled out. Flares were associated with discontinuation of IL-1 blockade before vaccination in four of the seven patients.

The retrospective design of this survey comes with a number of limitations. Due to this design, information on antibody titers is lacking as these are not routinely measured after vaccination. Therefore, we cannot draw any conclusions about the vaccine efficacy. Further, the small number of patients and the variety in diseases, age, medication and vaccines hampers conclusions on the safety of live-attenuated vaccines in general. Immunological characterization of patients before or after vaccination was not possible due to the retrospective design. The small number and the heterogeneity of the group precluded statistical analysis. The small number of patients included, however, reflects the reluctance of physicians to administer live-attenuated vaccines to patients using these biologicals.

A substantial number of patients in this series were vaccinated inadvertently through national vaccination programs, which has also led to a lack of follow-up data since patients were not monitored by their physician during and after vaccination. Live-attenuated vaccines cannot be considered entirely safe in patients using IL-1 or IL-6 blockade since up to three patients experienced an adverse event, while seven patients experienced a flare to some extent. This should be taken into consideration before administering live-attenuated vaccines in patients using IL-1 or IL-6 blockade.

At least one adverse event was considered to be caused by the micro-organism of the vaccine (patient 2, varicella). However, rash and in particular vesicles are also seen in 3% of healthy children after vaccination with varicella zoster vaccine [9]. Thus, the reported vesicles after vaccination might also be explained by a common vaccination reaction. In the two other patients with an adverse event it cannot be established with certainty that vaccination led to the symptoms of these patients, as pneumonia and diarrhea are common infections in childhood.

In our series, three of four MKD patients reported a flare after vaccination. It is already known that vaccination is a well-known trigger of fever episodes in MKD [10]. However, in these three patients the biological was stopped around the vaccination, as was the case in a child with another disorder who flared. The discontinuation might have contributed to the flares as well. Further, fever and rash are also quite common symptoms after vaccination in healthy children and adults. For example, fever is noted in 10–15% of children receiving varicella zoster vaccine [9]. After MMR vaccination 17% of healthy children and adults reported fever and 5% mentioned a rash [11].

Several studies have described the safety and efficacy of inactivated vaccines in patients using IL-1 blocking agents [12–15]. Two studies on canakinumab showed no difference in antibody titers between groups on canakinumab and subjects not on canakinumab [12, 13]. A study on anakinra also did not show a significant difference in antibody responses [14].

The data on disease flares and adverse events after vaccination are conflicting. In the phase-III trial of canakinumab in 109 CAPS patients, fifteen patients received influenza vaccination, five patients pneumococcal vaccination and one patient received MMR vaccine [16]. None of these patients reported an adverse event. Also in the study of 17 CAPS patients on canakinumab, no flares were described. Adverse events included predominantly upper respiratory tract infections. However, a recent study showed that CAPS patients treated with canakinumab reacted severely after pneumococcal vaccination [17]. Twelve of 18 patients who received pneumococcal immunizations developed vaccine reactions (fever, swelling, erythema, pain), usually within hours after vaccination. Reactions lasted up to 3 weeks. In two patients pneumococcal vaccination triggered CAPS reactivation with systemic inflammation.

## Conclusions

In conclusion, we have described a retrospective case series of seventeen patients who received live-attenuated vaccines while using IL-1 or IL-6 blocking medication. The current data are insufficient to draw any conclusions about the safety of these vaccines in patients using IL-1/IL-6 blockade. Therefore, more safety and efficacy data are needed before considering adaptation of guidelines. Until that time, physicians should still balance the risk of natural infection versus the risk of vaccination, including disease flares and other adverse events, for each individual patient.
